# Erratum to “TRPC1: The link between functionally distinct store-operated calcium channels” [Cell Calcium 42 (2007) 213–223]

**DOI:** 10.1016/j.ceca.2008.02.004

**Published:** 2008-10

**Authors:** Indu S. Ambudkar, Hwei Ling Ong, Xibao Liu, Bidhan C. Bandyopadhyay, Kwong Tai Cheng

**Affiliations:** Secretory Physiology Section, GTTB, NIDCR, NIH, Bethesda, MD 20892, USA

When this article was originally published the fourth author's name did not include the middle initial. It is printed correctly as above.

